# Interleukin-4 Receptor α Subunit Deficiency Alleviates Murine Intestinal Inflammation In Vivo Through the Enhancement of Intestinal Mucosal Barrier Function

**DOI:** 10.3389/fphar.2020.573470

**Published:** 2020-10-28

**Authors:** Ai Hertati, Shusaku Hayashi, Yudai Ogawa, Takeshi Yamamoto, Makoto Kadowaki

**Affiliations:** ^1^ Division of Gastrointestinal Pathophysiology, Institute of Natural Medicine, University of Toyama, Toyama, Japan; ^2^ Research Center for Biotechnology, Indonesian Institute of Sciences, Cibinong, Indonesia

**Keywords:** intestinal mucosal barrier, inflammatory bowel disease, interleukin-4 receptor, NADPH oxidase, reactive oxygen species, intestinal permeability

## Abstract

Disturbance of epithelial barrier function causes chronic intestinal inflammation such as inflammatory bowel disease. Several studies have reported that Th2 cytokines such as interleukin (IL)-4 and IL-13 play an important role in the regulation of intestinal barrier function. However, the precise role of the IL-4 receptor α subunit (IL-4Rα) in intestinal inflammation remains unclear. Thus, we used an experimental colitis model to investigate the role of IL-4Rα in intestinal inflammation. IL-4Rα-deficient (IL-4Rα-/-) mice and their littermate wild-type (WT) mice were used. Experimental colitis was induced by administration of 3% dextran sulfate sodium (DSS) in the drinking water for seven days. Treatment with DSS caused body weight loss, an increase in the disease activity index and histological abnormalities in WT colitis mice, all of which were significantly attenuated in IL-4Rα-/- colitis mice. Neutrophil infiltration in the colonic mucosa was reduced in IL-4Rα-/- colitis mice compared with WT colitis mice. NADPH oxidase 1 expression and reactive oxygen species production were increased in the colons of IL-4Rα-/- mice. Furthermore, elevated intestinal permeability induced by DSS treatment was suppressed in IL-4Rα-/- colitis mice. These results demonstrate that IL-4Rα-/- mice exhibit reduced susceptibility to DSS-induced colitis. Our present findings suggest that IL-4Rα deficiency enhances intestinal mucosal barrier function through the upregulation of NADPH oxidase 1-dependent reactive oxygen species production, thereby suppressing the development of intestinal inflammation.

## Introduction

The intestinal mucosa forms a selective barrier that simultaneously allows nutrient absorption and limits the uptake of antigens from the lumen (Quiros and Nusrat, 2019). Intestinal mucosal barriers include not only physical barriers, such as the mucus layer, glycocalyx and cell junctions, but also chemical barriers, such as antimicrobial peptides and proteins of the regenerating islet-derived three family, these barriers are regulated by intestinal environmental factors and cellular networks including epithelial cells, mesenchymal cells, immune cells and neuronal cells (Okumura and Takeda, 2017). Cytokines and their receptors are key players in cellular interactions in the intestine under both physiological and pathophysiological conditions (Friedrich et al., 2019). Breakdown of the intestinal mucosal barrier plays a pivotal role in the pathogenesis of intestinal immune-related disorders such as infectious colitis and inflammatory bowel disease (IBD) (Okumura and Takeda, 2017; Hayashi, 2020). Reductions in mucus production and antimicrobial peptide levels have been reported in IBD patients (Wehkamp et al., 2002; Swidsinski et al., 2007). Additionally, mice genetically deficient in mucosal barrier components showed high sensitivity to intestinal inflammation (Johansson et al., 2008).

Interleukin (IL)-4 and IL-13 are multifunctional and central cytokines in type two immune responses (Egholm et al., 2019). These cytokines signal through the IL-4 receptor (IL-4R) system, which consists mainly of the IL-4Rα subunit (LaPorte et al., 2008). IL-4Rα has been reported to express in various types of cells including epithelial cells (Takeda et al., 2010), tissue-resident macrophages (Jenkins et al., 2013) and neuronal cells (Lee et al., 2018). Several studies have provided evidences that IL-4 and IL-13 play a role in the regulation of intestinal mucosal barrier functions (Groschwitz and Hogan, 2009). For example, stimulation with IL-4 or IL-13 increased the permeability of human colonic epithelial monolayers (Zünd et al., 1996; Berin et al., 1999). IL-4- and IL-13-mediated intestinal epithelial barrier dysfunction was improved in mice lacking signal transducer and activator of transcription (STAT) 6, a downstream effector of IL-4R activation (Madden et al., 2002). Moreover, in ulcerative colitis (UC) patients, lamina propria mononuclear cells (LPMCs) in inflamed colonic mucosa produce large amounts of IL-13 compared with LPMCs in noninflammatory control patients (Heller et al., 2005). Furthermore, expression of IL-4Rα was detected in the colonic enterocytes of UC patients (Heller et al., 2005). These findings suggest that intestinal IL-4Rα mediates intestinal inflammatory responses. However, the role of IL-4Rα in intestinal mucosal barrier function and inflammation *in vivo* remains unclear.

In the present study, we investigated the role of IL-4Rα in intestinal mucosal inflammation using a model of dextran sulfate sodium (DSS)-induced colitis in IL-4Rα-deficient (IL-4Rα-/-) mice. DSS-induced intestinal inflammation was alleviated in IL-4Rα-/- mice through enhancement of intestinal mucosal barrier function in a manner dependent on NADPH oxidase 1 (NOX1)-derived reactive oxygen species (ROS).

## Materials and Methods

### Mice

IL-4Rα-/- mice (BALB/cJ-*Il4ra* ^*tm1Sz*^) on a BALB/c background (Noben-Trauth et al., 1997) were purchased from Taconic Biosciences (Rensselaer, NY, United States). To allow the use of littermate IL-4Rα-/- mice and wild-type (WT) mice in all experiments, mice with heterozygous deficiency of IL-4Rα were crossed, and the genotype of littermates was confirmed (Supplementary Figure S1A). All mice were housed in the experimental animal facility at the University of Toyama and were provided free access to food and water. In this study, 16 male mice in each normal group and 24 male mice in each colitis group were used at 10–12 weeks. All experiments were performed in accordance with the Guide for the Care and Use of Laboratory Animals of the National Institute of Health and the University of Toyama. The Animal Experiment Committee at the University of Toyama approved all animal care procedures and study protocols (authorization no. A2015INM-2 and A2018INM-3).

### Experimental Model of Colitis

Mice were treated with 3% DSS (36–50 kDa; MP Biomedicals, Santa Ana, CA, United States) in their drinking water for seven days (Hayashi et al., 2017). The body weight, stool consistency, and presence of blood in the stool were monitored daily to assess the severity of colitis. The disease activity index was calculated as the average of two parameters: diarrhea (0, normal; 1, soft stools; 2, loose stools; 3, mild diarrhea; 4, severe diarrhea) and blood in the stool (0, normal; 1, faint bleeding; 2, slight bleeding; 3, gross bleeding; 4, severe bleeding). The assay was terminated when weight loss reached 20% of initial body weight as the humane endpoint.

### Histological Analysis

The distal part of the colon was fixed in 4% paraformaldehyde, embedded in Tissue Freezing Medium (TBS, Durham, NC, United States), and sliced into 10 µm thick sections at −20°C using a cryostat microtome (Leica Microsystems, Nussloch, Germany). Sections were then routinely stained with hematoxylin and eosin (H&E). H&E-stained sections were scored for inflammation and crypt damage as previously described (Hayashi et al., 2014).

### Immunohistochemical Analysis

Immunohistochemical staining was performed according to the procedure described in previous reports (Lee et al., 2013). In brief, the mice were sacrificed seven days after DSS treatment, and the distal colons were excised and fixed by immersion in 4% paraformaldehyde for 2 h. After treatment with a 30% sucrose solution, tissue samples were embedded in OCT compound (Sakura Finetek, Tokyo, Japan). Frozen sections (30 μm) were soaked for 1 h in 0.3% Triton X-100 solution and incubated with normal donkey serum (1:10; Jackson ImmunoResearch Laboratories, West Grove, PA) for 1 h. Sections were incubated first with a rabbit anti-myeloperoxidase (MPO) antibody (1:200; Abcam, Cambridge, United Kingdom) or rabbit anti-IgG antibody (Wako, Osaka, Japan) for isotype control MPO antibody (Supplementary Figure S2) and then with Alexa Fluor 488-conjugated donkey anti-rabbit IgG (1:400; Jackson ImmunoResearch Laboratories). Sections were mounted with VECTASHIELD mounting medium containing DAPI (Vector Laboratories, Peterborough, United Kingdom). Immunostained sections were observed using a confocal laser scanning microscope (LSM780; Carl Zeiss, Oberkochen, Germany) and imaged. The number of MPO-positive cells in the colonic mucosa was determined using ImageJ software (NIH, Bethesda, MD, United States).

### RNA Extraction and Quantitative Real-time Polymerase Chain Reaction

The mRNA expression levels of various genes were measured in mouse colon samples as described previously (Hayashi et al., 2017). In brief, total RNA was extracted from the colons using Sepasol RNA I Super (Nacalai Tesque, Kyoto, Japan) according to the manufacturer’s instructions. Reverse transcription was performed using a PrimeScript RT Reagent Kit (Takara Bio, Otsu, Japan) and random primers. qPCR amplification was then performed using TB Green Premix EX Taq (Takara Bio) in a Takara TP800 thermal cycler (Takara Bio). The PCR conditions were as follows: 10 s at 95°C, followed by 40 cycles of 5 s at 95°C and 20 s at 60–63°C. Target mRNA levels were normalized to those of *Gapdh* as the internal control in each sample. The results are expressed as ratios relative to the average for the control group. The primer sequences are shown in Supplementary Table.

### Transcriptome Analysis

Transcriptome analysis was performed on mouse colon samples as described previously (Nagata et al., 2017). In brief, total RNA was extracted from the distal colons on day 7 after initiation of DSS treatment. mRNA was purified from the mixture of total RNA using an RNeasy Mini Kit (Qiagen, Crawley, United Kingdom), and mRNA was pooled from three to six mice per group (WT normal, WT colitis and IL-4Rα-/- colitis). Microarray analysis was performed using a GeneChip Mouse Gene 1.0 ST Array (Affymetrix, Santa Clara, CA, United States). The GeneChip was scanned with a GeneChip Scanner 3000 (Affymetrix), and gene expression was analyzed using GeneChip Analysis Suite Software (Affymetrix). Median centered and log2 transformed transcript levels are indicated by the following color code: blue, low; red, high. Furthermore, the data were analyzed GeneChip microarray data using Transcriptome Analysis Console (TAC; Thermo Fisher Scientific, Waltham, MA, United States), GeneSpring (Silicon Genetics, Redwood City, CA, United States) and Ingenuity Pathway Analysis (IPA; Ingenuity Systems, Redwood City, CA, United States, http://www.ingenuity.com) to extract significant genes, and identify the gene ontology and the canonical pathways associated with the differentially expressed genes.

### Measurement of Reactive Oxygen Species Production

ROS production was measured according to the procedure described in a previous report (Yokota et al., 2017). Whole colon tissues isolated from WT or IL-4Rα-/- mice were washed with ice-cold PBS, homogenized in ice-cold Krebs-HEPES buffer (pH 7.4) containing a protease inhibitor cocktail (Complete Mini; Roche, Mannheim, Germany), and centrifuged at 1,100 × g and 4°C for 15 minutes. The production of ROS (primarily superoxide) was measured with a chemiluminescence assay using L-012 (Wako, Osaka, Japan). Chemiluminescence was measured with a luminometer (Lumat LB 9507, Berthold Technologies, Germany). The level of ROS production is presented as arbitrary light units per minute per mg protein.

### *In Vivo* Intestinal Permeability Assay

Intestinal permeability was measured by determining the FITC-dextran concentration in the plasma of normal mice or DSS-induced colitis mice according to the procedure described in a previous study with slight modifications (Gupta et al., 2014). In brief, normal mice and colitis mice were fasted overnight. Mice were administered FITC-dextran (4 kDa; Chondrex, Redmond, WA, United States) (200 mg/kg body weight, po), while control mice were orally administered PBS (-). Four hours after oral gavage, mice were anesthetized, and blood was collected by cardiac puncture. Blood samples were centrifuged (2,500 × g, 10 minutes, 4°C), and plasma was collected. The fluorescence intensity in plasma samples was measured in black 96-well plates using a microplate reader (Tecan GENios; TECAN, Männedorf, Switzerland) at an excitation wavelength of 485 nm and emission wavelength of 535 nm. FITC-dextran concentrations were calculated from a standard curve.

### Statistical Analysis

The data are presented as the means ± SEMs. Statistical analyses were performed with Prism 8 (GraphPad Software, San Diego, CA) using one- or two-way ANOVA followed by Bonferroni’s multiple comparison test or an unpaired (two-tailed) t test with Welch’s correction. Values of *p* < 0.05 were considered to indicate significant differences.

## Results

### IL-4Rα-/- Mice Show Reduced Susceptibility to Dextran Sulfate Sodium-Induced Colitis

**FIGURE 1:**
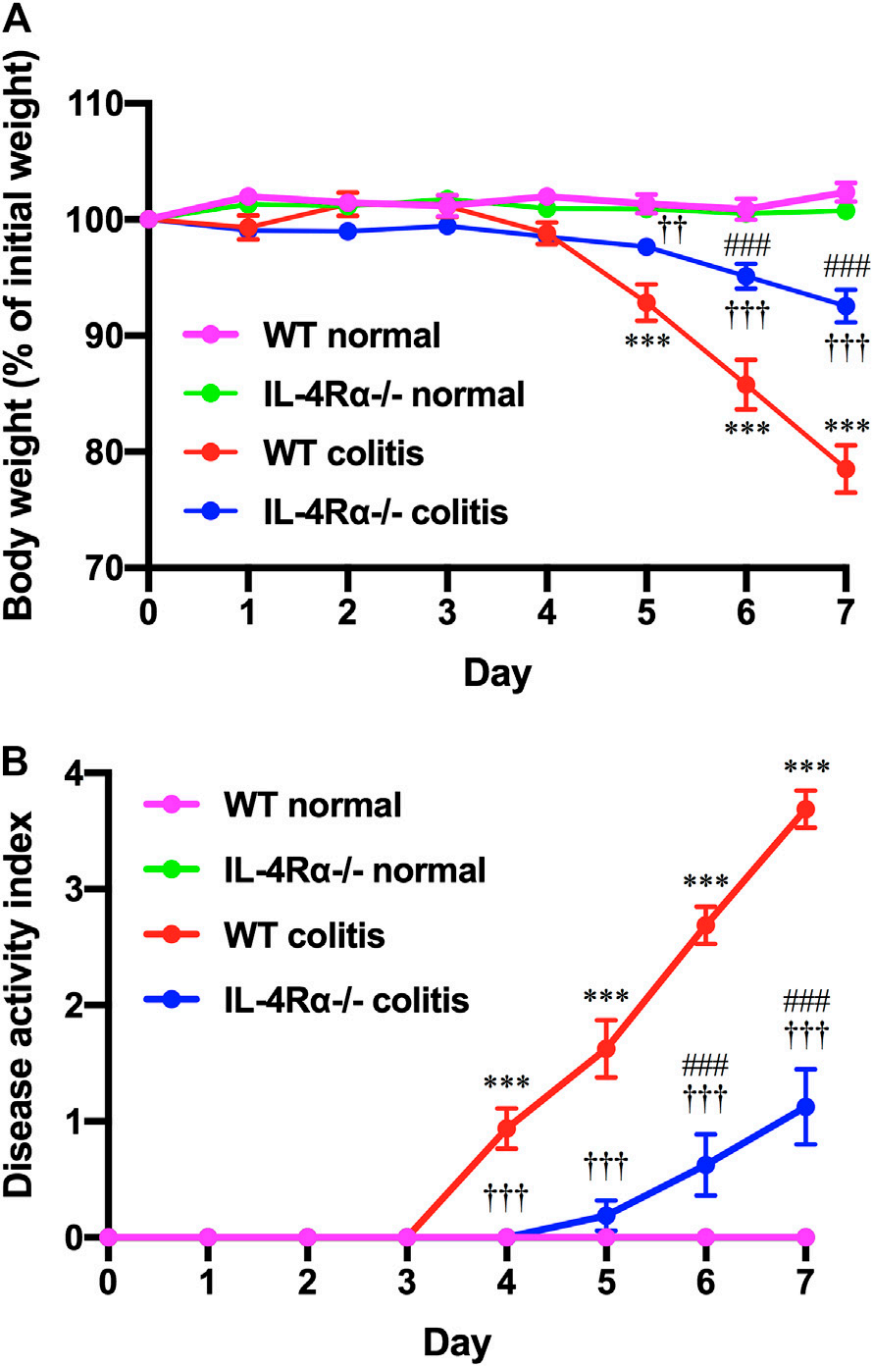
IL-4Rα-/- mice exhibit reduced susceptibility to DSS-induced colitis. Colitis was induced in WT and IL-4Rα-/- mice by daily treatment with a 3% DSS solution in the drinking water for seven days. **(A)** Body weight and **(B)** disease activity index are shown. The data are presented as the mean ± SEM values for eight mice and are representative of one of three independent experiments. ***p < 0.001 compared with WT normal mice. ††*p* < 0.01 and †††*p* < 0.001 compared with WT colitis mice. ###*p* < 0.001 compared with IL-4Rα-/- normal mice.

**FIGURE 2:**
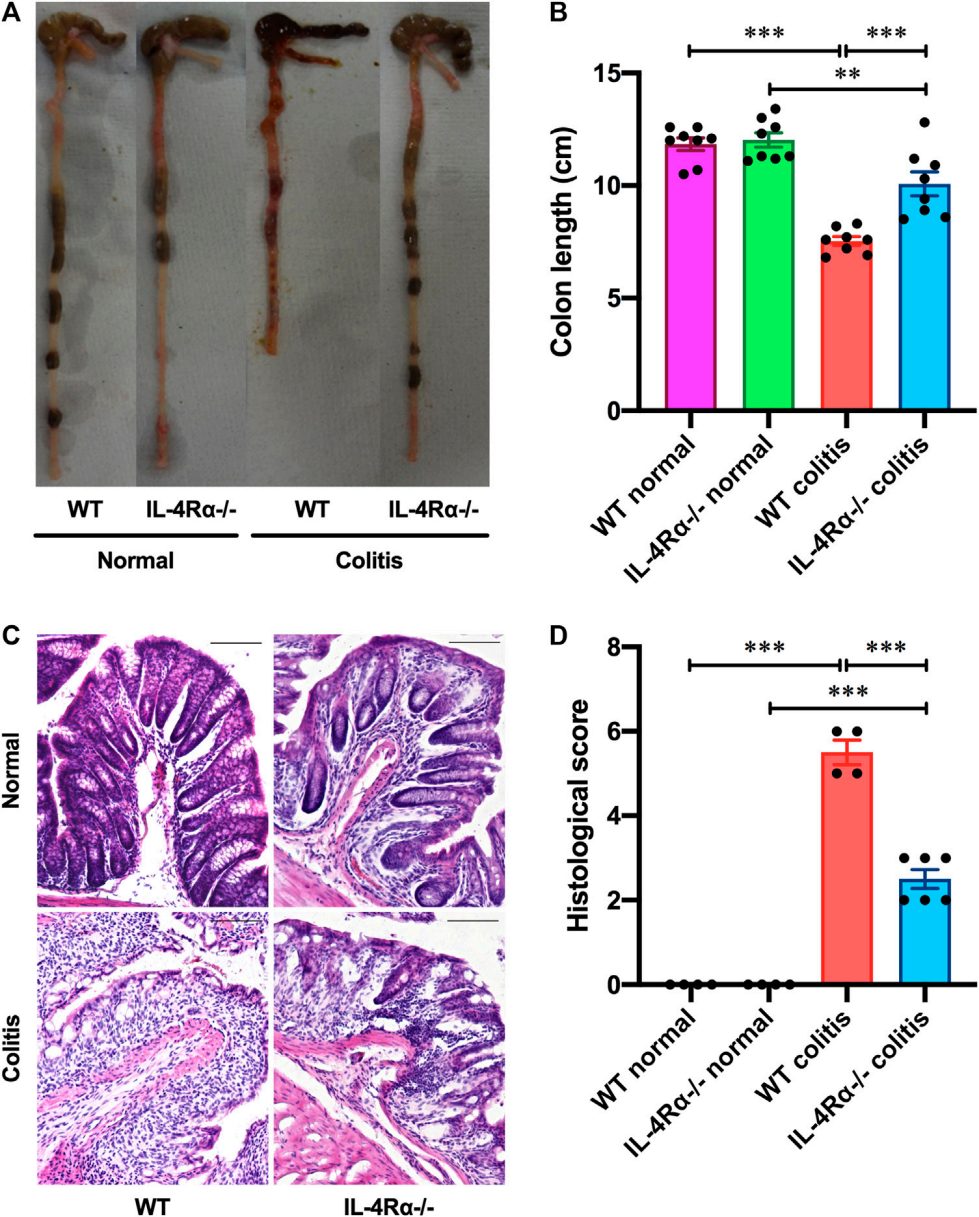
IL-4Rα-/- mice exhibit reduced susceptibility to DSS-induced colitis. Colitis was induced in WT and IL-4Rα-/- mice by daily treatment with a 3% DSS solution in the drinking water for seven days. **(A)** Macroscopic photographs of colons and **(B)** colon length measurements are shown. The data are presented as the mean ± SEM values for eight mice and are representative of one of three independent experiments. **p < 0.01; ***p < 0.001. **(C)** Representative images of H&E staining are shown. The scale bar represents 100 µm. **(D)** Histological scoring of DSS-induced colitis is shown. The data are presented as the mean ± SEM values for four to six mice. ***p < 0.001.

To determine the role of IL-4Rα in intestinal inflammation, we examined the development of DSS-induced acute colitis in WT and IL-4Rα-/- mice. On day 4 after DSS treatment via the drinking water, a decrease in body weight was observed in WT colitis mice but was significantly suppressed in IL-4Rα-/- mice. (Figure 1A; 78.5 ± 2.0% in WT colitis, 92.6 ± 1.4% in IL-4Rα-/- colitis mice on day 7; *p* < 0.001). The disease activity index was calculated as the average of the diarrhea score and rectal bleeding score and was significantly attenuated in IL-4Rα-/- colitis mice compared with WT colitis mice (Figure 1B; 3.7 ± 0.2 in WT colitis, 1.1 ± 0.3 in IL-4Rα-/- colitis on day 7; *p* < 0.001). Macroscopic observations of colons on day 7 showed that colon shortening occurred in WT colitis mice but was significantly alleviated in IL-4Rα-/- colitis mice (Figures 2A,B; 7.5 ± 0.2 cm in WT colitis, 10.1 ± 0.5 cm in IL-4Rα-/- colitis; *p* < 0.001). No difference was found in the colon length between WT and IL-4Rα-/- mice without colitis (normal, Figures 2A,B). Histological analysis by H&E staining showed that the colons of WT colitis mice exhibited a loss of epithelial integrity and crypt architecture (Figure 2C). In addition, submucosal edema occurred in the colons of WT colitis mice. These histological abnormalities were significantly ameliorated in the colons of IL-4Rα-/- colitis mice (Figures 2C,D; 5.5 ± 0.3 in WT colitis, 2.5 ± 0.2 in IL-4Rα-/- colitis; *p* < 0.001). We confirmed that the *Il-4rα* mRNA expression was totally depleted in the colon of IL-4Rα-/- mice (Supplementary Figure S1B).

**FIGURE 3:**
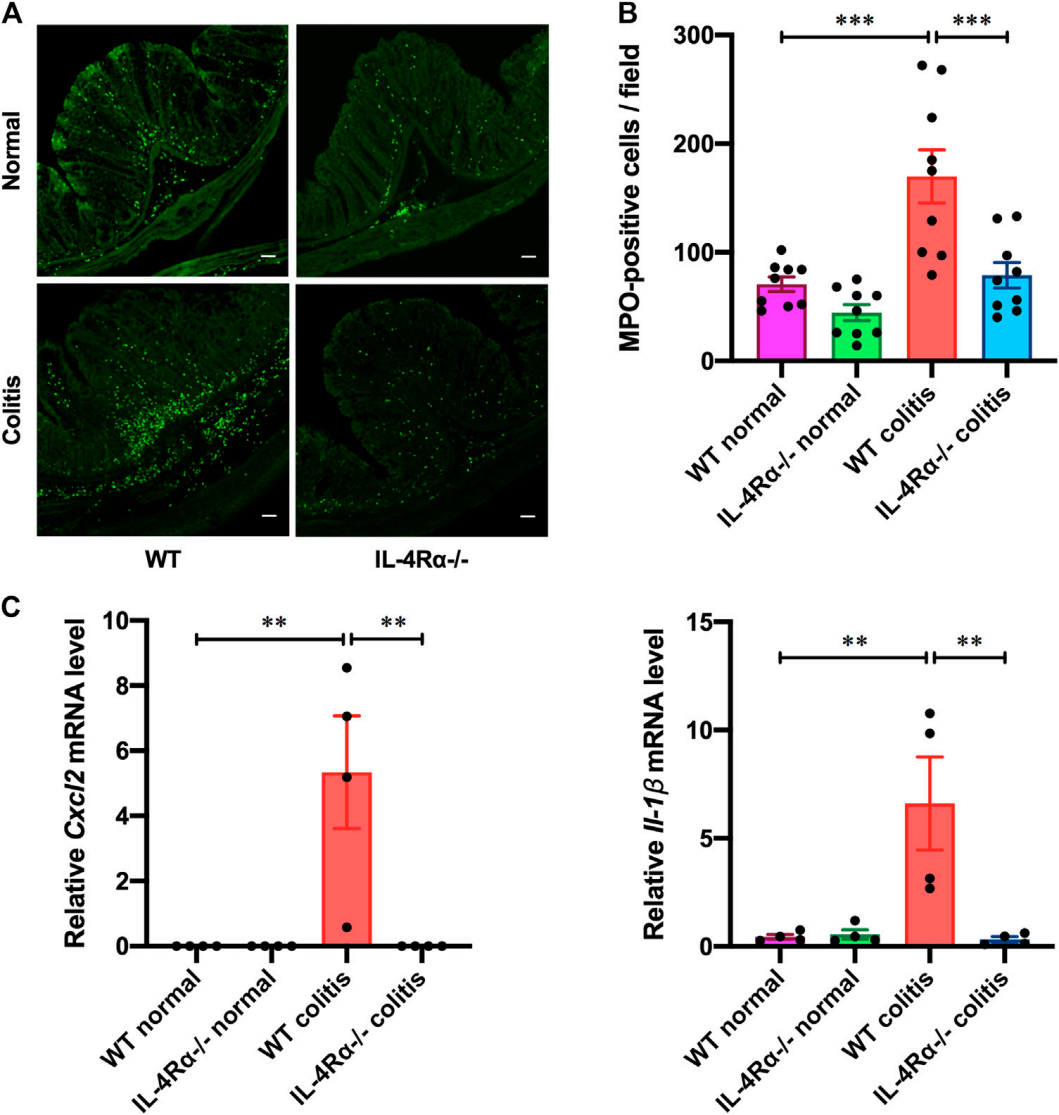
IL-4Rα-/- mice exhibit a low inflammatory response to DSS-induced colitis. Colitis was induced in WT and IL-4Rα-/- mice by daily treatment with 3% DSS solution in the drinking water for seven days. **(A)** Confocal micrographs of the colonic mucosa of colitis mice show staining of MPO-positive cells. The scale bar represents 50 µm. **(B)** The number of MPO-positive cells in the colonic mucosa is shown. The data are presented as the mean ± SEM values for nine mucosae from three mice. ***p < 0.001. **(C)** DSS-induced changes in the mRNA expression level of *Cxcl*2 and *Il-1β* in the colons of WT and IL-4Rα-/- mice. The data are presented as the mean ± SEM values for four mice and are representative of one of two independent experiments. *p < 0.05; **p < 0.01.

Next, we evaluated the immunoreactivity of MPO to assess the distribution of neutrophils in the colonic mucosa of colitis mice seven days after initiation of DSS treatment. The number of infiltrated neutrophils in the colonic mucosa of IL-4Rα-/- colitis mice was dramatically reduced compared to that in WT colitis mice (Figures 3A,B; 169.9 ± 24.5 in WT colitis, 78.9 ± 11.7 in IL-4Rα-/- colitis; *p* < 0.001). The mRNA expression level of *Cxcl2* and *Il-1β* in the colons of WT colitis mice was markedly elevated on day 7 (Figure 3C; *Cxcl2*; 0.0 ± 0.0 in WT normal, 5.3 ± 1.7 in WT colitis; *p* < 0.01, *Il-1β*; 0.4 ± 0.1 in WT normal, 6.6 ± 2.1 in WT colitis; *p* < 0.01). However, the increase in *Cxcl2* and *Il-1β* mRNA expression was significantly suppressed in the colons of IL-4Rα-/- colitis mice (Figure 3C; *Cxcl2*; 5.3 ± 1.7 in WT colitis, 0.5 ± 0.3 in IL-4Rα-/- colitis; *p* < 0.05, *Il-1β*; 6.6 ± 2.1 in WT colitis, 0.3 ± 0.1 in IL-4Rα-/- colitis; *p* < 0.01). These results clarify that the development of DSS-induced colitis is suppressed in IL-4Rα-/- mice compared with WT mice.

### IL-4Rα Deficiency Increases NADPH Oxidase 1-Dependent Reactive Oxygen Species Production in the Mouse Colon

**FIGURE 4:**
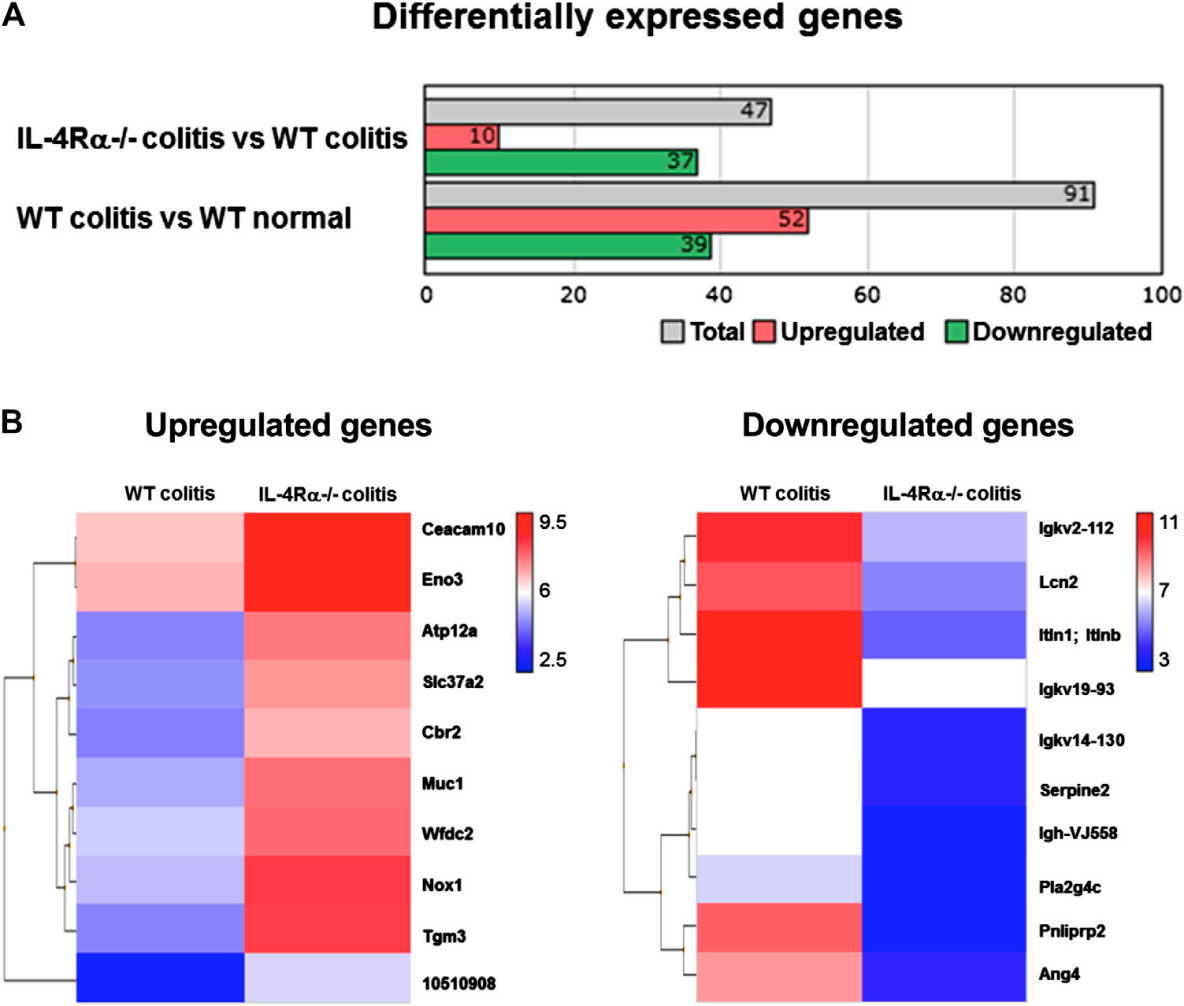
Transcriptomic analysis shows that IL-4Rα-/- mice exhibit a high level of *Nox1* mRNA expression in DSS-induced colitis model. Microarray data sets of WT normal, WT colitis (day 7) and IL-4Rα-/- colitis (day 7) mice are analyzed using Transcriptomic Analysis Console (TAC) Software. **(A)** Differentially expressed genes whose expression levels are more than four-fold or less than quarter in comparison between IL-4Rα-/- colitis and WT colitis or WT colitis and WT normal. **(B)** Heat map shows top 10 upregulated (left) and downregulated (right) in IL-4Rα-/- colitis mice compared with WT colitis mice. The color range represents log2-transformed fold changes. Each row corresponds to each gene, and each column corresponds to each group. Genes were sorted using hierarchical clustering based on Euclidean distance and average linkage, as shown in the dendrogram on the left side of the heatmap. The 10 gene names are displayed on the right side of the heatmap.

**FIGURE 5:**
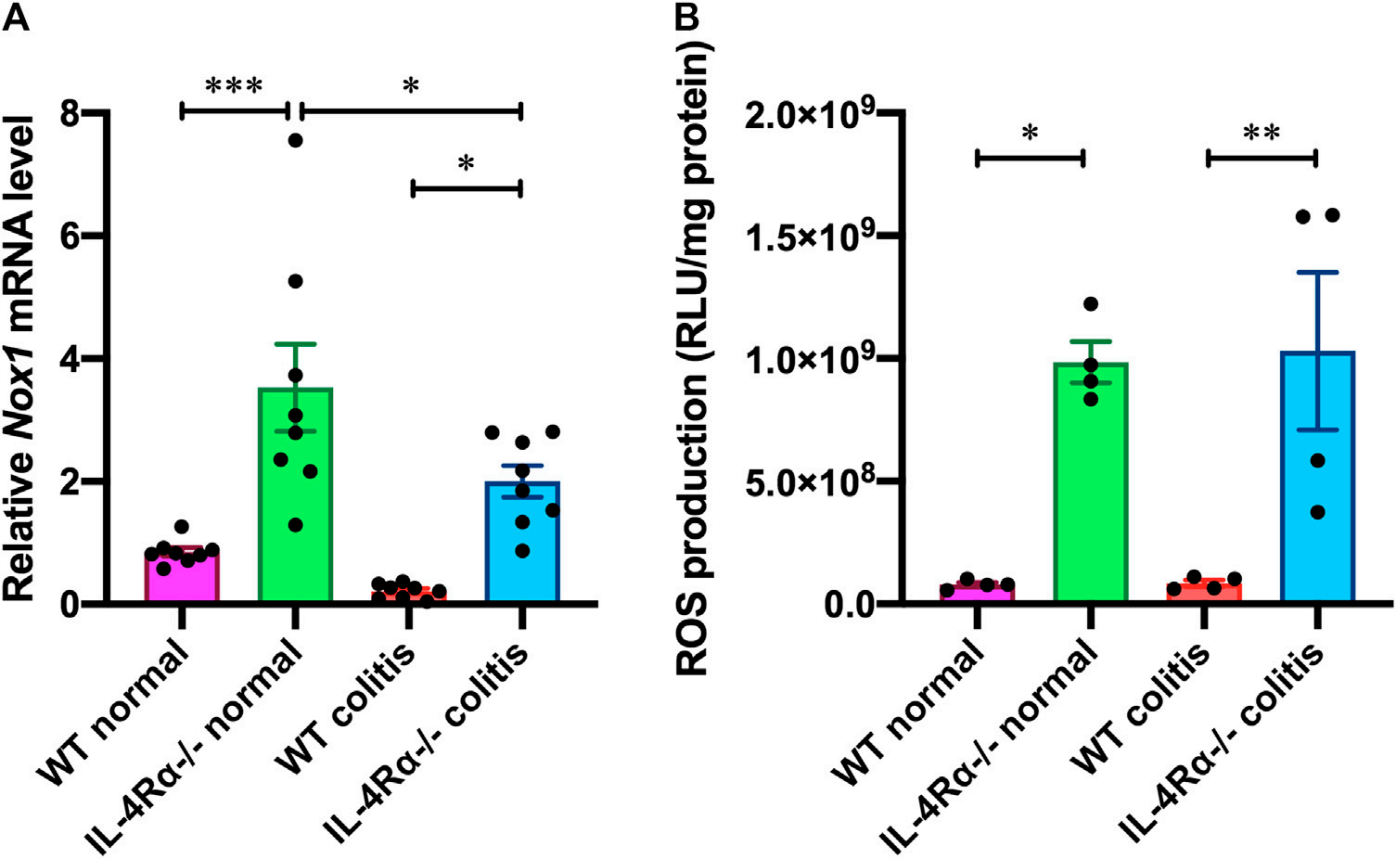
IL-4Rα deficiency increases NOX1-dependent ROS production in the colon of mouse. **(A)** The mRNA expression level of *Nox1* in the colons of WT and IL-4Rα-/- mice. The data are presented as the mean ± SEM values for eight mice. *p < 0.05; ***p < 0.001. **(B)** ROS production (RLU, relative luminescence units) in the colons of WT and IL-4Rα-/- mice. The data are presented as the mean ± SEM values for four mice and are representative of one of two independent experiments. *p < 0.05; **p < 0.01.

To investigate changes in colonic gene expression levels between WT colitis and IL-4Rα-/- colitis mice, we performed transcriptome analysis on colonic tissues obtained on day 7 after initiation of DSS treatment. The differential expression analysis using TAC showed that the expression level of 91 genes was altered by DSS treatment and 47 genes were differentially expressed between IL-4Rα-/- colitis and WT colitis mice (Figure 4A). Among 47 genes, 10 genes were upregulated and 37 genes were downregulated in IL-4Rα-/- colitis mice compared with WT colitis mice. Furthermore, the top 10 upregulated and downregulated genes in IL-4Rα-/- colitis mice compared to WT colitis mice were shown in the heat map (Figure 4B). To determine the biological significance of these genes, we performed GeneSpring and IPA. However, GeneSpring and IPA did not show any significant canonical pathway. Thus, among the differentially expressed genes, we focused on *Nox1* expression because NOX1 has been reported to contribute to the maintenance of intestinal homeostasis (Lambeth and Neish, 2014). To verify the transcriptome analysis results, we conducted qPCR to measure the mRNA expression level of *Nox1* in colitis mice. Consistent with the transcriptome analysis result, the qPCR analysis results showed that the *Nox1* mRNA expression in the colons of IL-4Rα-/- colitis mice was significantly higher than that in the colons of WT colitis mice (Figure 5A; 0.21 ± 0.04 in WT colitis, 2.00 ± 0.26 in IL-4Rα-/- colitis; *p* < 0.05). Furthermore, the depletion of IL-4Rα resulted in significant 4.2-fold increase in the mRNA expression level of *Nox1* in the colons of normal mice (Figure 5A; 0.85 ± 0.07 in WT normal, 3.53 ± 0.71 in IL-4Rα-/- normal; *p* < 0.05). To evaluate the contribution of IL-4Rα to NOX1 activity, we measured ROS production in the colonic tissues of mice. The production of ROS was significantly increased in the colons of IL-4Rα-/- normal mice compared with WT normal mice (Figure 5B; 0.7 × 10^8^ ± 0.1 × 10^8^ RLU/mg protein in WT normal, 9.8 × 10^8^ ± 0.8 × 10^8^ RLU/mg protein in IL-4Rα-/- colitis; *p* < 0.05). We also observed significant increase in the production of ROS in the colons of IL-4Rα-/- colitis mice compared to WT colitis mice, (Figure 5B; 0.8 × 10^8^ ± 0.1 × 10^8^ RLU/mg protein in WT colitis, 10.3 × 10^8^ ± 3.2 × 10^8^ RLU/mg protein in IL-4Rα-/- colitis; *p* < 0.01). These findings indicate that IL-4Rα deficiency enhances ROS production in the colonic mucosa through the upregulation of NOX1 expression.

### IL-4Rα Deficiency Enhances Intestinal Barrier Function *in Vivo*

**FIGURE 6:**
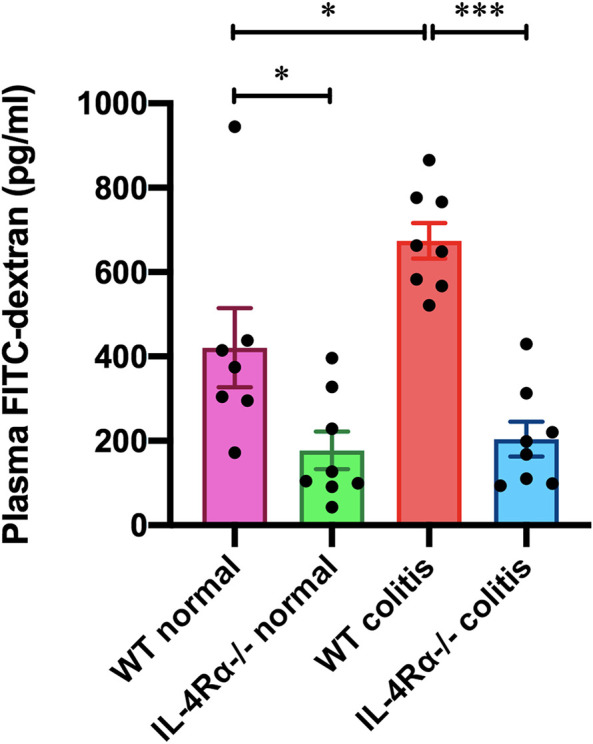
IL-4Rα deficiency enhances intestinal barrier function *in vivo*. The concentration of FITC-dextran in the plasma of WT and IL-4Rα-/- mice is shown. Plasma samples were collected from WT and IL-4Rα-/- mice on day 0 (normal) or day 7 (colitis) after initiation of DSS treatment. The data are presented as the mean ± SEM values for seven to eight mice. *p < 0.05; ***p < 0.001.

We measured the level of 4 kDa FITC-dextran in the plasma to investigate intestinal permeability in the colons of mice. The FITC-dextran level in the plasma of WT colitis mice was significantly elevated on day 7 (Figure 6). The elevated FITC-dextran level was markedly decreased in the IL-4Rα-/- colitis mice (Figure 6; 673.9 ± 42.1 pg/ml in WT colitis, 204.0 ± 41.5 pg/ml in IL-4Rα-/- colitis; *p* < 0.001). Interestingly, the level of plasma FITC-dextran was significantly lower in IL-4Rα-/- normal mice than in WT normal mice (Figure 6; 420.6 ± 93.6 pg/ml in WT normal; 177.3 ± 44.8 pg/ml in IL-4Rα-/- normal; *p* < 0.05).

## Discussion

Our results indicate that the development of DSS-induced colitis is alleviated in IL-4Rα-/- mice through enhancement of NOX1-derived ROS production in the colon.

IBD is a chronic inflammatory disorder of the intestine comprising Crohn’s disease and UC (Kaser et al., 2010). Genetic, clinical and experimental studies have shown that IBD is a multifactorial disease in which a genetically inadequate host response to environmental factors leads to a breakdown of the intestinal mucosal barrier (Maloy and Powrie, 2011). Maintenance of the intestinal mucosal barrier is mediated by a tissue-specialized cellular network via the signaling pathways of various cytokine receptors (Friedrich et al., 2019). Breakdown of the cellular network regulated by cytokines and their receptors is recognized as the pathogenic mechanism underlying disorders of chronic intestinal inflammation, such as IBD. Indeed, many IBD risk loci have been found in regions of genes encoding cytokines or their downstream signaling mediators (Liu et al., 2015). An IL-13-dependent abnormal Th2 cell response has been observed in the colonic mucosa of IBD patients (Heller et al., 2005). IL-4 and IL-13 have also been reported to play critical pathogenic roles in animal models of colitis (Mizoguchi et al., 1999; Heller et al., 2002). IL-4Rα was strongly expressed in the colonic tumors of murine colitis-associated cancer (CAC) model in WT mice, and the number and size of colonic tumors were significantly reduced in IL-4Rα-/- CAC mice through the suppression of epithelial tumor proliferation due to lack of IL-4Rα-/- signaling, however there was no information about the inflammation in this previous study (Koller et al., 2010). Although the involvement of IL-4Rα in the disruption of epithelial barrier function in IBD patients has been suggested (Heller et al., 2005), no direct evidence has revealed the role of IL-4Rα in mediating intestinal inflammation.

To investigate the role of IL-4Rα in the intestinal mucosal inflammation, we used a model of DSS-induced colitis. Mice deficient in IL-4Rα exhibited obvious amelioration of symptoms of DSS-induced colitis such as diarrhea, rectal bleeding, colon shortening, and destruction of the colonic mucosal structure. In addition, DSS-induced neutrophil infiltration in the colonic mucosa was decreased in IL-4Rα-/- mice. Neutrophil infiltration in the mucosa is mediated by specific chemoattractants such as CXCL1, CXCL2 (also known as macrophage inflammatory protein 2; MIP2) and CXCL8 (Farooq et al., 2009). Notably, CXCL2-overexpressing transgenic mice showed that the increased number of MPO-positive neutrophils in the colonic lamina propria of normal and DSS-treated mice (Ohtsuka and Sanderson, 2003). Intestinal mucosal neutrophils, which infiltrate the inflamed mucosa of IBD patients and animals in experimental colitis models, produce various cytokines that trigger further inflammatory responses (Baumgart and Carding, 2007). Among these cytokines, IL-1β exhibits increased production in the model of DSS-induced colitis (Melgar et al., 2005; Hayashi et al., 2017). We previously reported that the expression level of IL-1β in the colonic mucosa was positively correlated with the disease severity of DSS-induced colitis in mice (Hayashi et al., 2017). Furthermore, IL-1β secreted from infiltrated neutrophils in the colonic mucosa contributes to the pathogenesis of colitis (Wang et al., 2014). In the present study, the infiltration of neutrophils and the upregulation of CXCL2 and IL-1β observed in colonic tissues of colitis mice were significantly suppressed in the IL-4Rα-/- mice. These findings strongly demonstrate that IL-4Rα-/- mice exhibit reduced susceptibility to inflammatory responses in the intestine.

We also measured *Il-4* and *Il-13* mRNA expression in the colonic tissues of normal and colitis mice (Supplementary Figure S3). The mRNA expression of *Il-4* was significantly lower in IL-4Rα-/- colitis mice than in WT colitis mice. IL-4 deficient mice have been reported to show amelioration of DSS-induced colitis (Stevceva et al., 2001), suggesting the pathogenic role of IL-4 in DSS-induced colitis model. However, the roles of IL-4 in DSS-induced colitis model are still controversial because protective roles of IL-4 have been also reported (Kim and Chung, 2013). In the present study, there was no difference in *Il-4* mRNA expression between WT normal mice and WT colitis mice. Furthermore, the depletion of IL-4Rα resulted in 2.4-fold increase in mRNA expression level of *Il-13* in the colons of IL-4Rα-/- normal mice. Since IL-13 increases intestinal permeability dependent on IL-4Rα/STAT6 activation, elevated IL-13 in IL-4Rα-/- normal mice is assumed to have no effect on intestinal permeability due to lack of IL-4Rα in the present study. Although IL-4 and IL-13 are recognized as key cytokines for IL-4Rα-mediated mucosal barrier function (Groschwitz and Hogan, 2009), further detailed studies are required to understand the role of IL-4 and IL-13 in the intestinal mucosal barrier function *in vivo*.

Colonic tissues of IL-4Rα-/- mice exhibited high *Nox1* mRNA expression under both steady and inflammatory conditions. NOX1 is a NADPH oxidase, and is a major source of ROS in nonphagocytic cells (Lambeth and Neish, 2014). Recent studies have reported that NOX1-dependent ROS production in intestinal epithelial cells plays important roles in maintaining intestinal homeostasis (Lambeth and Neish, 2014). Defective ROS production due to mutations in the *NOX1* gene has been suggested to be associated with an increased risk of very early onset IBD (Hayes et al., 2015; Stenke et al., 2019). IL-10-deficient mice are well known as a model of spontaneous colitis (Kühn et al., 1993) because IL-10 secreted from intestinal macrophages plays a pivotal role in the regulation of intestinal homeostasis during host defense (Maloy and Powrie, 2011; Hayashi, 2020). Mice with combined deficiency of NOX1 and IL-10 exhibited colitis symptoms earlier than IL-10-deficient mice (Tréton et al., 2014). Furthermore, mutant mice that generate low intestinal levels of ROS developed more severe DSS-induced colitis than WT mice (Aviello et al., 2019). In the present study, we observed that deficiency of IL-4Rα enhanced the production of ROS in colonic tissues of normal mice. These findings suggest that IL-4Rα deficiency in mice enhances ROS generation due to upregulation of Nox1 expression in the colon, resulting in the suppression of colitis development. However, pathogenic roles of NOX1-derived ROS in colitis have also been reported. Administration of apocynin, an antioxidant and a nonselective NADPH inhibitor, suppressed the inflammatory response in a model of DSS- and tumor necrosis factor-α-induced colitis (Mouzaoui et al., 2014; Ramonaite et al., 2014). NOX1-deficient mice showed reduced susceptibility to trinitrobenzene sulfonic acid-induced colitis through downregulation of proinflammatory cytokine expression in macrophages (Yokota et al., 2017; Makhezer et al., 2019). These conflicting results in models of colitis using NOX1-deficient mice may be due to the differences in the colitis models and the responsible cells in which NOX1 functions.

Since NOX1 is highly expressed in intestinal epithelial cells (Szanto et al., 2005; Valente et al., 2008), epithelial NOX1-derived ROS have been reported to play pivotal roles in the control of intestinal epithelial integrity. Intestinal epithelial NOX1-derived ROS generation and subsequent ROS signaling have been demonstrated to orchestrate intestinal mucosal wound repair after injury (Leoni et al., 2013). ROS produced by epithelial NOX1 contribute to the proliferation and differentiation of colonic epithelial cells by modulating the PI3K/AKT/Wnt and Notch1 signaling pathways (Coant et al., 2010). Furthermore, NOX1-mediated cellular ROS production is required for collective migration of epithelial cells, because defective directional migration and altered cell-cell contact were observed in cells with loss of NOX1 function (Khoshnevisan et al., 2020). Intestinal epithelial cells cover the outermost surface of the mucosa and interface with the lamina propria to form the intestinal mucosal barrier (Quiros and Nusrat, 2019). Thus, NOX1-dependent ROS production in intestinal epithelial cells is considered an important property for maintaining intestinal barrier integrity. These findings led to the hypothesis that IL-4Rα mediates intestinal mucosal barrier function through the regulation of NOX1-dependent ROS production. To address this hypothesis, we evaluated intestinal barrier function in IL-4Rα-/- mice as assessed by intestinal permeability, because intestinal permeability is strongly related to intestinal mucosal barrier function (Luissint et al., 2016). IL-4Rα-/- mice showed a reduction in intestinal permeability to 4 kDa FITC-dextran, suggesting that the intestinal barrier function of IL-4Rα-/- mice may be strengthened by upregulation of NOX1-derived ROS production. However, further studies are needed to clarify the mechanism by which IL-4Rα signaling regulates NOX1 expression in intestinal epithelial cells.

Given the findings of our present study, we conclude that IL-4Rα deficiency enhances NOX1-dependent ROS production and intestinal mucosal barrier function, resulting in the suppression of colitis development. Although further detailed studies are required, the role of IL-4Rα in the intestinal inflammation revealed here may improve the understanding of the pathogenesis of refractory intestinal diseases such as IBD. Recently, sustained clinical remission has been recognized as an ideal therapeutic goal for IBD (Neurath and Travis, 2012). To achieve long-term remission, repairing injured mucosa and rebuilding the barrier integrity in the intestinal mucosa is required after induction of remission. Our findings may lead developing a novel therapeutic strategy aimed at maintaining remission in IBD through the regulation of ROS production mediated by IL-4Rα signaling.

## Data Availability Statement

The raw data supporting the conclusions of this manuscript will be made available by the authors, without undue reservation, to any qualified researcher.

## Author Contributions

AH and SH designed the research and analyzed the data; AH and YO performed experiments; TY provided technical support; AH and SH drafted the manuscript; All authors edited and reviewed the manuscript; and SH and MK acquired funding.

## Funding

This research was supported by JSPS KAKENHI grants JP17KK0166 and 18K06698 (SH) and JP20K07098 (MK) from the Ministry of Education, Culture, Sports, Science and Technology of Japan and by the Kobayashi Foundation (SH).

## Conflict of Interest

The authors declare that the research was conducted in the absence of any commercial or financial relationships that could be construed as a potential conflict of interest.
