# Supplementary material for: Interleukin-4 Receptor α Subunit Deficiency Alleviates Murine Intestinal Inflammation In Vivo Through the Enhancement of Intestinal Mucosal Barrier Function
Source: Front Pharmacol. 2020 Oct 28;11:573470. doi: 10.3389/fphar.2020.573470 (PMC7656058; doi:10.3389/fphar.2020.573470)
Supplement: Supplementary file 1 [file DataSheet1_v1.docx]

Supplementary Material

**Supplementary Method**

**Genotyping**

To determine genotyping of littermates, PCR was performed using primer pairs specific for exon 7 and the *neo* gene in a single reaction according to the previous report (Noben-Trauth et al., 1997). DNA was extracted from the tails of mice using DirectPCR Lysis Reagent (Viagen Biotech, Los Angeles, CA, USA) according to the manufacturer’s instructions. PCR amplification was performed using MightyAmp DNA polymerase (Takara Bio). The PCR conditions were as follows: initial denaturation at 98°C for 2 minutes, followed by 25 cycles of amplification (98°C for 10 seconds, 60°C for 15 seconds and 68°C for 1 minute). A portion of the PCR mixture was electrophoresed on 2% agarose gel containing 0.1% ethidium bromide in Tris-borate-EDTA buffer and photographed.

**RNA extraction and quantitative real-time PCR (qPCR)**

The mRNA expression levels of *Il-4rα*, *Il-4* and *Il-13* were measured in mouse colon samples as described previously (Hayashi et al., 2017). Target mRNA levels were normalized to those of *Gapdh* as the internal control in each sample. The results are expressed as ratios relative to the average for the control group. The primer sequences are shown in Supplementary Table.

**Supplementary Table. Primer sequences for qPCR**

|  | **Primer sequence, 5’-3’** | |
| --- | --- | --- |
| **Gene** | **Forward** | **Reverse** |
| *Gapdh* | TGACCACAGTCCATGCCATC | GACGGACACATTGGGGGTAG |
| *Cxcl2* | ACCCCACTGCGCCCAGACAGAA | AGCAGCCCAGGCTCCTCCTTTCC |
| *Il-1β* | CTGTGTCTTTCCCGTGGACC | CAGCTCATATGGGTCCGACA |
| *Il-4* | GGTCTCAACCCCCAGCTAGT | GCCGATGATCTCTCTCAAGTGAT |
| *Il-13* | GGATATTGCATGGCCTCTGTAAC | AACAGTTGCTTTGTGTAGCTGA |
| *Il-4rα* | ACGTGGTACAACCACTTCCA | TGGCTTCGGGTCTGCTTATC |
| *Nox-1* | AGTAGGTGTGCATATGGGTGT | ACCAGCCAGTTTCCCATTGT |

## Supplementary Figures


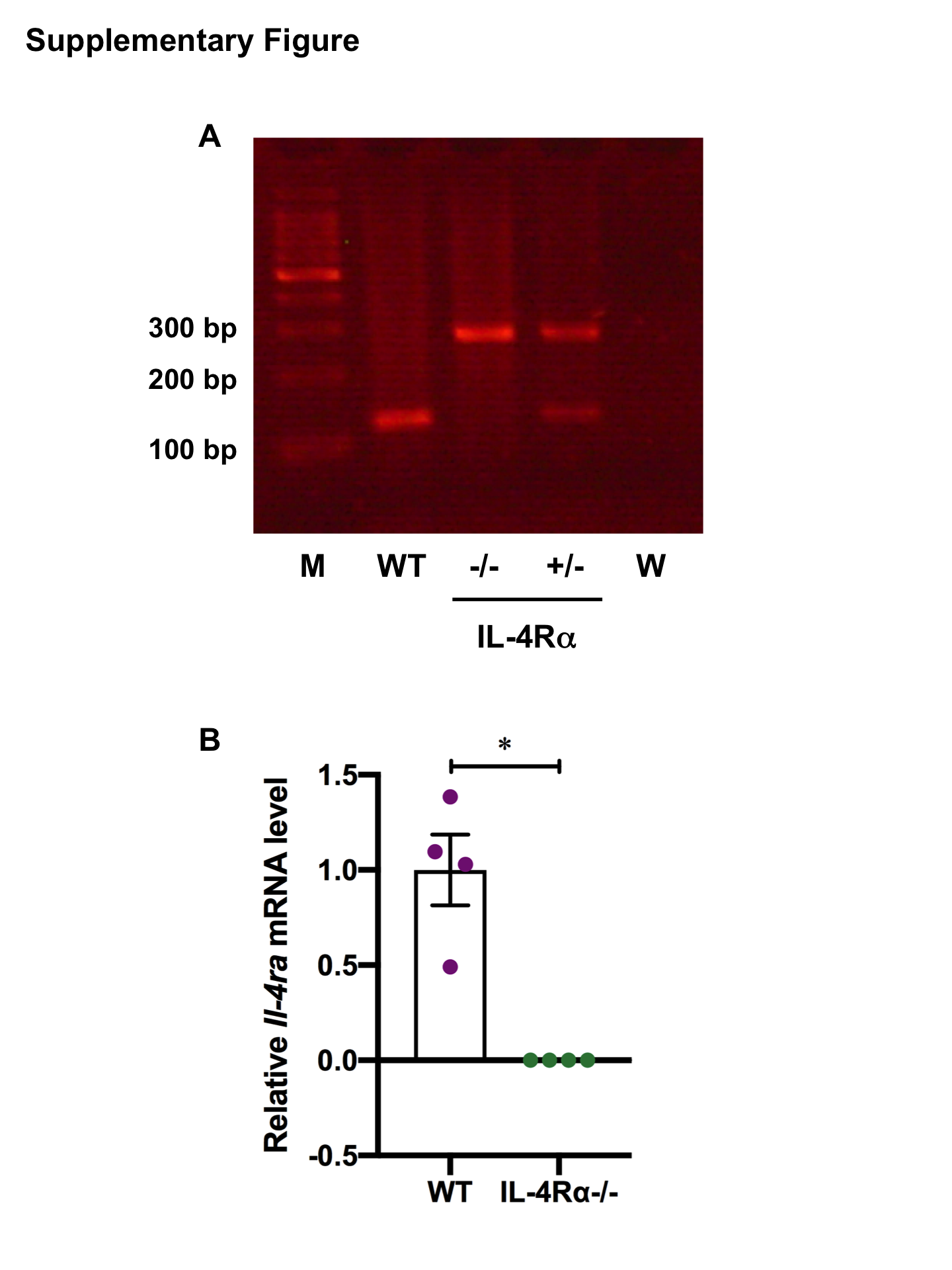


**Supplementary Figure 1.** (A) Representative images of genotyping are shown. PCR products reveal a 125 bp band in WT mice, a 280 bp band in IL-4Rα-/- mice, and 125 bp and 280 bp bands in IL-4Rα-hetero-deficient (IL-4Rα+/-) mice. Water (W) was amplified as negative control template. M, molecular mass markers. (B) The mRNA expression level of *Il-4rα* in the colons of WT and IL-4Rα-/- normal mice. The data are presented as the mean ± SEM values for 4 mice. *p<0.05.

**Supplementary Figure 2.** (A) Representative images of isotype control IgG staining for MPO antibody are shown. The scale bar represents 100 µm.


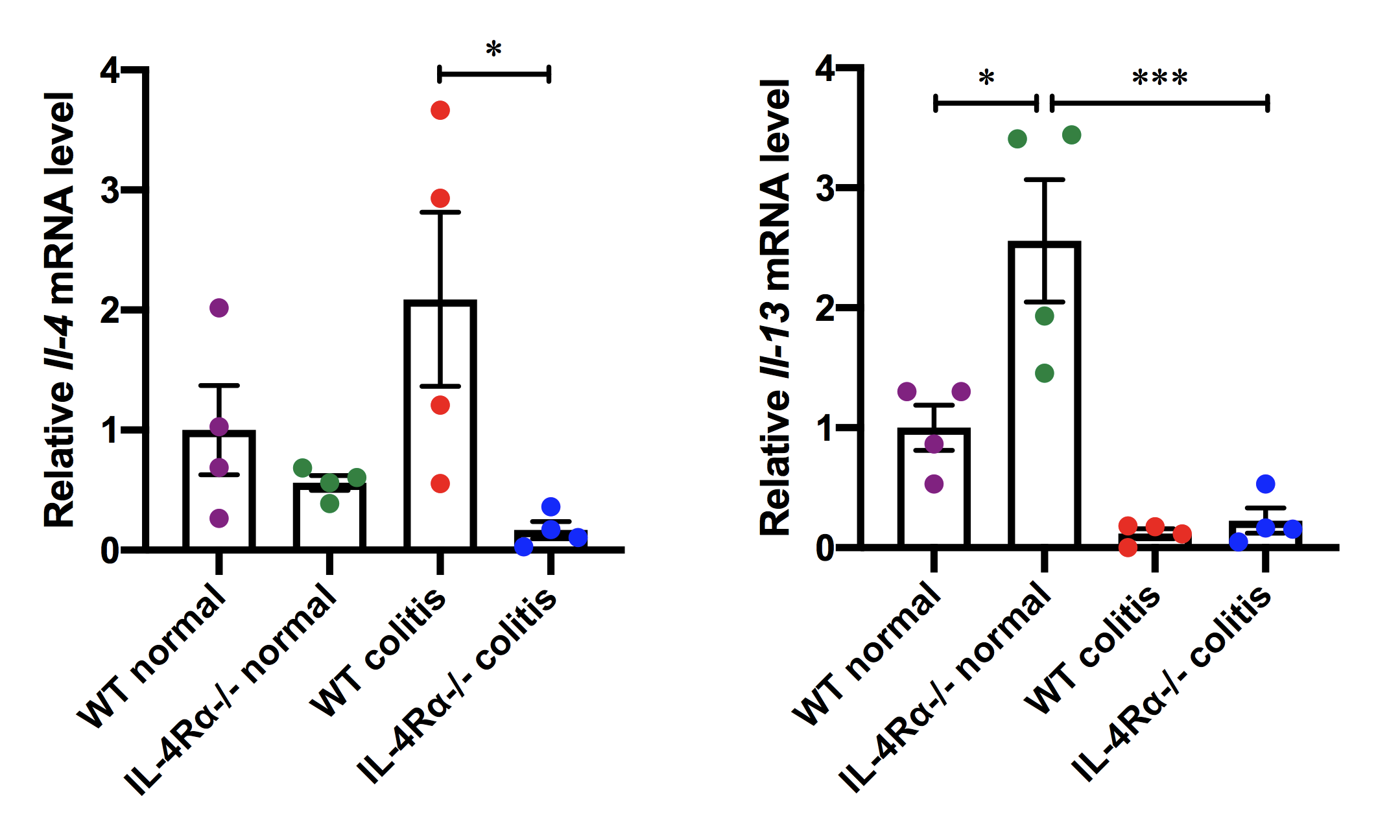


**Supplementary Figure 3.** DSS-induced changes in the mRNA expression level of *Il-4* and *Il-13* in the colons of WT and IL-4Rα-/- mice. The data are presented as the mean ± SEM values for 4 mice. *p<0.05; **p<0.01; ***p<0.001.
